# The sustainability of the healthcare: a cross-sectional study among Italian JCI-accredited hospitals

**DOI:** 10.1093/intqhc/mzag056

**Published:** 2026-05-07

**Authors:** Pier Mario Perrone, Martina Lauria, Fabrizio Tediosi, Silvana Castaldi

**Affiliations:** Department of Clinical Sciences and Community Health, University of Milan, Milan 20122, Italy; Department of Pathophysiology and Transplantation, University of Milan, Milan 20122, Italy; Quality Department, Humanitas Clinical and Research Hospital, Rozzano, MI 20089, Italy; Department of Pathophysiology and Transplantation, University of Milan, Milan 20122, Italy; Department of Biomedical Sciences for Health, University of Milan, Milan 20122, Italy; Quality Unit, Foundation IRCCS Ca’ Granda Ospedale Maggiore Policlinico, Milan 20122, Italy

## Abstract

**Background:**

The health sector is characterized by dual aspects in relation to climate change and its impact on human health. To consider firstly the treatment of resulting health problems; and secondly the representation of environmental impact as a primary cause. Consequently, a number of certification bodies have indicated their intention to incorporate sustainability criteria into their certification processes. The objective of this study is to analyze the present sustainability practices of JCI-accredited hospitals in Italy.

**Methods:**

The cross-sectional study was conducted using a 15-question questionnaire that was disseminated to all Italian JCI-accredited facilities. The 15 questions were structured according to the five domains outlined in the Global Green and Healthy Hospitals. These questions were both open questions regarding organizational challenges, achievements, and future strategies related to sustainability. In addition, there were closed questions for data on specific practices with multiple-choice formats.

**Results:**

A response rate of 95.2% was obtained, with a geographical distribution showing a prevalence of facilities in northern Italy (55%). Statistically significant differences were not observed between Northern, Central and Southern Italy with regard to years of accreditation, number of beds, presence of sustainability committees or presence of environmental risk assessments in the risk plan. The analysis of the GHI’s five domains reveals an average of approximately 60% of the responses indicating the presence of figures responsible for environmental sustainability, as well as for the analysis of environmental risks within the different company risk plans. With regard to the presence of a sustainability committee, 75% of the facilities indicated that they had one. In addition, the findings reveal that a significant proportion, close to (55%) of facilities have adopted strategies to mitigate waste generation within the supply chain, with 85% of facilities that adopt strategies reporting the implementation of specific measures to reduce waste.

**Conclusion:**

The issue of sustainability is becoming increasingly central to healthcare organizations, especially those accredited by the JCI. This is being addressed through the establishment of intra-company committees and the incorporation of environmental considerations into company procedures and regulations. This development signifies a preliminary step towards a paradigm shift within the Italian healthcare system, with the intention of incorporating environmental impact considerations into its accreditation requirements.

## Introduction

Handling Editor: Rosa Sunol

Climate change is widely recognized as one of the most significant challenges of the 21st century, with far-reaching implications not only on the ecosystems and economies but also on huma health. Its health-related impacts are increasingly evident, disproportionately affecting vulnerable populations and exacerbating existing health inequities [1].

In this context, the healthcare sector faces a fundamental paradox: while its core mission is to protect and promote health, it significantly contributes to environmental degradation through high levels of energy consumption, greenhouse gas emissions, and waste generation—both biomedical and general [2–7].

Hospitals and healthcare facilities are particularly resource-intensive institutions. Their environmental footprint stems from energy-intensive operations, extensive supply chains, and complex clinical and logistical infrastructures [8]. Recent analyses estimate that healthcare systems are responsible for nearly 5% of global carbon emissions, with hospitals accounting for a substantial share of this total [9, 10]. As a consequence, there is a growing recognition of the need to re-orient healthcare practices and systems towards sustainability. This transformation entails reducing unnecessary care, redesigning clinical pathways with lower environmental impact, and enhancing the resource efficiency and climate resilience of healthcare facilities [11–14]. However, advancing sustainability in healthcare must be pursued without compromising accessibility, equity, and quality of care [15–17]. Financial and organizational constraints—especially in publicly funded systems—require a pragmatic and a balanced approach, ensuring that sustainability goals are integrated into the broader mandate of health system performance.

In response to these imperatives, sustainability has gained increased prominence within international health governance frameworks. The 2030 Agenda for Sustainable Development, adopted by the United Nations in 2015, explicitly recognizes the interdependence of health, environmental integrity, and sustainable development. Among its guiding principles is the “5Ps” framework—People, Planet, Prosperity, Peace, and Partnership—highlighting the need for integrated and cross-sectoral action [18].

Reflecting this global shift, the Joint Commission International (JCI) introduced in 2024 a new section entitled *Global Health Impact* (GHI) within its evidence-based accreditation standards [19]. This section, comprises five standards and 22 indicators that may be adapted to the context of the individual accredited facility, establishing a structured framework for embedding environmental sustainability into hospital operations.

The GHI standards promote the monitoring and decarbonization of resource use, encourage the mitigation of environmental impacts across supply chains, and aim to strengthen the climate resilience of accredited facilities. As of 1 January 2025, all JCI-accredited institutions are required to comply with these sustainability standards in order to maintain their accreditation status.

Despite this development, the Italian National Health System (NHS) has yet to adopt environmental sustainability as a formal criterion in its national accreditation or performance assessment processes. In this context, JCI-accredited hospitals in Italy represent a unique and early-testing ground for the operationalization of sustainability standards within the country’s hospital sector. These institutions may serve as exemplars or laboratories of innovation, offering valuable insights into the feasibility, challenges, and enabling factors associated with implementing environmental sustainability in healthcare settings.

The objective of this study is to examine the current sustainability practices of JCI-accredited hospitals in Italy, assessing the extent to which these hospitals adhere to the five domains of the GHI through compliance with the newly introduced GHI standards, identifying the main challenges encountered in their implementation, and evaluating the possibility of developing and introducing opportunities for improvement and expansion.

## Methods

### Study design and setting

This cross-sectional study was conducted to assess the current sustainability practices of all hospitals in Italy accredited by the Joint Commission International (JCI) as of July 1st, 2024. A custom-designed questionnaire was developed by a team of JCI experts and administered to all 21 JCI-accredited healthcare facilities. The survey was distributed electronically in July 2024, with a follow-up reminder sent approximately 20 days later to maximize participation. JCI accreditation is an internationally recognized standard for healthcare quality and patient safety. In Italy, as of the date of this study, 21 healthcare facilities had obtained JCI accreditation.

### Participants

The questionnaire was distributed via personalized email to the managers responsible for quality of care at each JCI-accredited ­hospital. All invited participants had been directly involved in their institution’s JCI accreditation process within the past 5 years. Respondents were given the option to complete the survey online or request a Word version of the questionnaire for offline compilation.

### Study variables

The survey instrument was informed by international frameworks and guidelines, including the World Health Organization’s *Health Care’s Climate Footprint* (2018) and the United Nations Environment Programme’s *Safe Management of Wastes from Healthcare Activities* (2018). It consisted of 15 questions structured around the five domains outlined in the Global Green and Healthy Hospitals (GGHH) agenda, adapted to the JCI context:

1. Governance, tracking, and reporting—Monitoring of environmental objectives and designation of sustainability leadership.
2. Employee engagement and empowerment—Promotion of sustainable behaviors through staff education and awareness initiatives.
3. Use of environmental resources—Management of energy, water, and waste to reduce environmental impact.
4. Procurement and supply chain—Implementation of environmentally responsible purchasing policies.
5. Infrastructure and service resilience—Consideration of environmental risks in planning and infrastructure design.

The questionnaire combined closed-ended and open-ended questions:

- Closed-ended questions captured structured data on specific practices (e.g., use of renewable energy, sustainable procurement policies), with multiple-choice formats allowing for multiple selections.
- Open-ended questions encouraged detailed responses on organizational challenges, achievements, and future strategies related to sustainability.

Additionally, background data were collected, including the number of beds, year of JCI accreditation, and geographic location of the hospitals. For geographic classification, the NUTS (Nomenclature of Territorial Units for Statistics) framework was applied, with Northern Italy comprising both the North-West and North-East regions. This was undertaken to circumvent the potential for reclassification based on regional healthcare systems or social characteristics, which would have resulted in discrepancies in comparisons between areas. The objective was to ensure the classification was as precise as possible. In this sense, arbitrary classifications based on geographical boundaries such as rivers or mountains, which do not provide scientific evidence to ensure a correct division, were avoided.

To ensure confidentiality and reduce potential biases, all questions were phrased in a neutral and non-invasive manner. Participation was voluntary, and informed consent was obtained prior to questionnaire completion.

Moreover, in order to guarantee the content validity and reliability of the questionnaire, a multi-step expert review process was undertaken.

Initially, two JCI experts reviewed the JCI manual, and on this basis, the questionnaire items were developed in alignment with the five Global Health Impact (GHI) standards. Subsequently, the draft questionnaire was circulated to an independent panel of five additional JCI experts who had not been involved in the initial development phase, including a physician, a nurse, a healthcare administrator, a quality and patient safety professional, and a health management expert.

Each expert was tasked with independently interpreting the questionnaire items in order to assess clarity, relevance and consistency with the intended standards. This was followed by a structured group discussion involving all seven experts, the aim of which was to resolve discrepancies, refine item wording and reach consensus on the final version of the questionnaire.

### Data analysis

Descriptive statistical analyses were conducted on the quantitative data. Categorical variables were reported as absolute frequencies and percentages. Conversely, quantitative variables were characterised as mean and standard deviation, or as median and interquartile range, contingent upon the distribution of the data. Comparative analyses were performed using Fisher’s exact test or chi-squared test for qualitative variables. For quantitative variables, the Shapiro test was employed in order to assess the normality of the distribution. The Wilcoxon or t-student test was then used, as appropriate.

All open-ended responses were analyzed using a thematic coding approach. For each open-ended survey question, the coding was performed by a multidisciplinary team of JCI experts with experience in quality improvement and sustainability in healthcare.

The analysis followed three main steps. First, responses were independently reviewed and grouped into thematic categories (initial coding). Second, recurring themes were identified by counting the frequency of each category across responses. Third, dominant themes were highlighted and qualitatively interpreted to extract key insights.

A shared coding framework (codebook) was developed iteratively during the initial coding phase to ensure consistency in theme definitions. Coding decisions were discussed within the expert team, and discrepancies were resolved through consensus. Given the expert-driven and consensus-based nature of the process, formal inter-coder reliability statistics were not calculated. Responses were categorized either by domain (e.g. professional roles or organizational units) or by thematic characteristics (e.g. types of challenges or innovations). For questions involving organizational actors, responses were grouped into:

- Strategic directorates (e.g., CEOs, general management),
- Health directorates (e.g., medical and nursing leadership),
- Non-health personnel (e.g., administrative or technical staff).

Given the study’s focus on a specific subset of Italian hospitals—those with JCI accreditation—the results may not be generalizable to the broader national healthcare context.

All the statistical analyses were conducted with the R software, release 4.4.2.

## Results

### Structural and organizational characteristics

Table 1 presents the structural characteristics of the JCI-accredited hospitals included in this study. Geographically, the majority of institutions (*n* = 11; 55%) are located in Northern Italy, followed by six (30%) in Central Italy and three (15%) in the South. A marked predominance of private institutions was observed across all regions, with private facilities accounting for 72.7% in the North, 66.7% in the Center, and 100% in the South.

**Table 1:** Structural features of JCI-accredited hospital.

	North (*N* = 11)	Center (*N* = 6)	South (*N* = 3)	
Institution type				
Private (%)	8 (72.7)	4 (66.7)	3 (100)	*P*-value = .8084
Public (%)	3 (23.3)	2 (33.3)	0 (0)	
Environmental risk in Risk plan				
Yes (%)	9 (81.8)	3 (50)	1 (33.3)	*P*-value = .2265
No (%)	2 (18.2)	3 (50)	2 (66.7)	
Sustainability committee				
Yes (%)	3 (23.3)	1(16.7)	1 (33.3)	*P*-value = 1
No (%)	8 (72.7)	5 (83.3)	2 (66.7)	
N° of Beds (median [IQR])	311 [469]	299 [402.5]	97 [43.5]	*P*-value = .1173
Years of Accreditation (median [IQR])	17 [12]	6 [6.2]	10 [2.5]	*P*-value = .3133

Regarding the integration of environmental risk into institutional risk management plans, a greater proportion of facilities in the North (81.8%) reported such integration, compared to 50% in the Center and 33.3% in the South (*P* = .2265). However, no statistically significant differences emerged among regions. The presence of a sustainability committee was infrequent across all areas, with only 23.3% of facilities in the North, 16.7% in the Center, and 33.3% in the South reporting such a body.

Hospitals in the North had a higher median number of beds (311 [IQR: 469]) and longer accreditation history (17 years [IQR: 12]) compared to those in the Center (299 beds; 6 years) and the South (97 beds; 10 years). Despite observable differences, none reached statistical significance.

### Governance and monitoring (GHI standard 1)

As shown in Table 2, 60% of institutions reported having a designated figure responsible for sustainability-related projects. Among these, 20% reported directly to the General Director or CEO, while others reported to technical or clinical leadership positions.

**Table 2:** Governance, monitoring and reporting: monitoring of environmental targets and appointment of sustainability leaders.

	*N* = 20
Is there a person in charge of sustainability projects?	
Yes	12 (60%)
No	7 (35%)
NA	1 (5%)
To whom in hierarchy	
University Teaching Hospital, report to University Department Head	2 (10%)
Reports to General Management/CEO	4 (20%)
Reports to CFO	1 (5%)
Report to Medical director	3 (15%)
Report to Technical Office Director	2 (10%)
Do you have a sustainability committee?	
Yes	3 (15%)
No	15 (75%)
Missing data	2 (10%)
Numbers and composition	
RSPP (Health and Safety Officer)	2 (10%)
Energy Manager/Chief Sustainability Officer	2 (10%)
Human Resources Management	3 (15%)
General Manager	3 (15%)
Clinical Engineering	2 (10%)
Facility Manager	2 (10%)
Medical Directorate	1 (5%)
Quality Manager	3 (15%)
Procurement and Logistics Manager	3 (15%)
How do you measure your sustainability progress?	
No	7 (35%)
No, but it is a work in progress.	8 (40%)
Yes, there is an indicator system	5 (25%)

Only 15% of facilities had a formal sustainability committee, and committee composition varied widely. Participating members included representatives from Human Resources, Quality, Procurement and Logistics, Clinical Engineering, and General Management, reflecting a multidisciplinary but inconsistent approach.

In terms of performance measurement, 40% of hospitals were in the process of implementing monitoring tools, 25% had a structured indicator system in place, and 35% reported the absence of any such tools.

### Staff engagement and sustainability culture (GHI standard 2)

As illustrated in Table 3, 65% of institutions had implemented initiatives to promote sustainability culture. Common strategies included the dissemination of internal newsletters (40%), staff training programs (10%), and workshops or seminars (20%).

**Table 3:** Employee involvement and empowerment: promotion of sustainable practices and education through staff training and awareness-raising.

	*N* = 20
Do you promote the spread of sustainability culture within your organisation?	
Yes	13 (65%)
No	7 (35%)
1 interventions	8 (40%)
2 interventions	4 (20%)
3 interventions	1 (5%)
Yes, we organize workshops and seminars on sustainability.	4 (20%)
Yes, we have a mandatory training program for all staff.	2 (10%)
Yes, we regularly communicate through internal newsletters about sustainable initiatives.	8 (40%)
Other	5 (25%)
All staff	13 (65%)
Only leaders	4 (20%)
Doctors, Nurses, and Healthcare Assistants (OSS)	2 (10%)
External partners and/or suppliers	3 (15%)
Other (please describe briefly)	3 (15%)

Interventions targeted multiple staff levels in most cases (65%), and external stakeholders such as suppliers were involved in 15% of cases. However, only a minority of facilities implemented more than two sustainability initiatives, suggesting room for broader engagement.

### Resource efficiency and environmental impact (GHI standard 3)

As illustrated in Table 4, 90% of facilities had implemented at least one measure to reduce energy consumption, with 65% reporting more than one intervention. The most commonly adopted strategies included the use of LED lighting (55%), HVAC system upgrades (40%), and the integration of renewable energy sources, such as solar panels (45%).

**Table 4:** Use of environmental resources: efficient management of energy, water and waste to minimise environmental impact.

	*N* = 20
Measures implemented to reduce energy consumption	
0 measure	2 (10%)
1 measures	4 (20%)
1 < 5 measures	13 (65%)
>5 measures	1 (5%)
Yes, we have installed LED lighting systems throughout the hospital.	11 (55%)
Yes, we have upgraded our heating, ventilation, and air conditioning systems to improve efficiency.	8 (40%)
Yes, we use energy-efficient medical equipment.	2 (10%)
Yes, we have implemented an energy management system to monitor and reduce energy consumption.	8 (40%)
Yes, we have installed solar panels to integrate renewable energy into our energy needs.	9 (45%)
Yes, we have programmed lighting and climate control systems to turn off automatically when not needed.	5 (25%)
Yes, we are using cogeneration systems to efficiently produce both electricity and thermal energy.	4 (20%)
Other	6 (30%)
In the risk plan, do you consider environmental risks and scenarios that could affect service delivery?	
Yes	13 (65%)
No	7 (35%)
Land use, building design and regulatory environment	10 (50%)
Planning for infrastructure protection and resilience	9 (45%)
Planning for the provision of essential clinical care services	9 (45%)
Environmental protection and ecosystem adaptations	7 (35%)
Programs implemented for waste management and reduction	
1 measures	5 (25%)
1 < 5 measures	15 (75%)
Yes, we have implemented a recycling program for paper, plastic and glass. Yes, we have taken measures to reduce the use of disposable materials.	12 (60%) 5 (25%)
Yes, we have a hazardous and medical waste management program.	16 (80%)
Yes, we have started a composting program for organic waste.	2 (10%)
Yes, we have established policies to reduce food waste in the hospital cafeteria.	3 (15%)
Yes, we have introduced recycling bins in all areas of the hospital.	14 (70%)
Other	3 (15%)

Incorporation of environmental risk into risk management plans was reported by 65% of facilities. Key areas addressed included land use (50%), infrastructure resilience (45%), and essential clinical service continuity (45%).

Waste management practices were widely adopted: 80% had medical waste management programs, 60% implemented recycling programs, and 70% introduced hospital-wide recycling bins. Composting and food waste reduction programs were reported less frequently.

### Sustainable procurement (GHI standard 4) and integration of sustainability in strategic planning (GHI standard 5)

As illustrated in Table 5, sustainability criteria were considered in supplier selection by 55% of hospitals. Among these, 20% directly evaluated suppliers internally, and 15% relied on certified suppliers. A majority (85%) reported initiatives aimed at waste reduction in the supply chain, particularly through improved materials management (60%) and monitoring mechanisms (50%). Packaging management was less frequently targeted (25%). Moreover, regarding the integration of sustainability in strategic planning, the 80% of hospitals reported prioritizing sustainability in their operational planning. Sustainability efforts were particularly focused on environmental aspects (30%) and clinical service optimization (40%), while attention to energy and technological aspects was less prominent (5% each).

**Table 5:** Supply chain sustainable purchasing practices and Infrastructure Resilience.

	*N* = 20
Do you consider sustainability criteria when selecting suppliers?	
Yes	11 (55%)
No	9 (45%)
Supplier direct evaluation by institute*	4 (20%)
Suppliers certified	3 (15%)
Other	6 (30%)
Have you taken steps to reduce waste in your supply chain?	
Yes	17 (85%)
No	3 (15%)
1	7 (35%)
2–3	13 (65%)
4–5	0 (0%)
Materials and stock management	12 (60%)
Packaging management	5 (25%)
Monitoring	10 (50%)
Has any attention been paid to the issue of sustainability in your hospital?	
Yes	16 (80%)
No	4 (20%)
In which areas?	
Environment	6 (30%)
Energy	1 (5%)
Technology	1 (5%)
Medical Services Optimization	8 (40%)

## Discussion

### Statement of principal findings

This study provides the first systematic assessment of environmental sustainability practices among JCI-accredited hospitals in Italy. The findings reveal a heterogeneous landscape: while many institutions have initiated sustainability-oriented actions, substantial gaps persist in governance, staff engagement, systematic monitoring, and the strategic integration of sustainability principles into core planning and operations. These gaps have direct operational implications. Limited governance and weak strategic integration constrain hospitals’ ability to prioritize sustainability actions, allocate resources coherently, and ensure accountability over time. Similarly, limited staff engagement, particularly beyond senior management, reduces the likelihood that sustainability becomes embedded in routine clinical and operational decision-making, where many high-impact behaviors occur.

In recent years, environmental sustainability has emerged as a critical concern in healthcare, driven by growing awareness of the sector’s substantial contribution to climate change and its downstream impacts on public health. Consequently, there is an increasing necessity for such facilities to pay greater attention to their own processes, in both management and delivery [20–22].

Despite this, the absence of a robust national regulatory framework in Italy has limited the systemic integration of environmental sustainability within healthcare governance. In this vacuum, individual healthcare institutions retain discretion over the structure and scope of their sustainability efforts, resulting in considerable variability across facilities.

In this respect, the study demonstrates that the composition of the sustainability committees is characterized by significant heterogeneity [4, 23–25], which is contingent on the intrinsic characteristics of the structures in question [26, 27]. This heterogeneity is evident in terms of structural organization, and with respect to the services offered.

Nevertheless, the majority of facilities appear to demonstrate a significant degree of attention regarding initiatives aimed at ­communicating with staff through internal newsletters and the organization of seminars and workshops on environmental sustainability issues.However, our findings suggest that communication efforts are not consistently matched by structured mechanisms for staff participation in sustainability planning, implementation, and monitoring. This disconnect implies that sustainability may remain a top-down initiative, limiting translation into everyday practices and reducing the likelihood of identifying operational changes with high environmental impact.

This dualism, although not explicitly articulated, could be interpreted as a notable emphasis on the issue, whether in terms of regulatory frameworks, requests from certifying bodies, or the branding of healthcare companies. However, it could also be argued that the application of this dualism within the corporate context is not equally prioritized, given the substantial investment of time and human resources required. It is imperative to acknowledge the significance of staff involvement in the evaluation of interventions, particularly in the context of initiatives that facilitate the achievement of specific indicators [28]. The absence of such involvement may result in the absence of requests for structural changes, which can have substantial ramifications for sustainability. This, in turn, can lead to a considerable augmentation in implementation and management costs. This result directly supports the need for broader staff engagement and distributed leadership, as hospitals are unlikely to achieve sustained improvements if sustainability remains confined to a limited group of decision-makers.

As Salas has demonstrated, the decarbonization of healthcare systems is an intricate and multifaceted process [29]. The establishment of boundaries within the system is imperative, encompassing the production and transportation of medical supplies, the movement of patients and personnel, the utilization of energy in healthcare facilities, the investment portfolios of healthcare organizations, and associated domains. In this context, the presence of individuals with diverse expertise and professional backgrounds can offer a range of perspectives, which is undoubtedly advantageous for a healthcare company.

However, it should be noted that health professional represents only one aspect of environmental sustainability. Other considerations include direct intervention in the services offered and supplies used [12, 30, 31]. In this regard, comprehensive accounting requires consideration of the entire life cycle of healthcare products and processes, as well as the allocation of all associated carbon footprint contributions. It is also necessary to consider the cost in terms of pollution, energy consumption and waste in healthcare realities.

In this regard, the study’s findings are encouraging, with a focus on energy efficiency strategies, primarily based on the utilization of recyclable energy sources and the implementation of low-waste lighting and air-conditioning systems. Additionally, the study explores the management of supply chains and the environmental impact of suppliers, with a particular emphasis on supply chain waste management and monitoring activities.

The analysis also reveals major inconsistencies. Although many institutions express intentions to adopt low-impact technologies, few report investments in long-term solutions, such as cogeneration systems or high-efficiency medical equipment. The limited uptake of these high-impact interventions reflects both financial constraints and a lack of strategic vision, despite increasing availability of guidance from national and international ESG (environmental, social, and governance) frameworks.

Interestingly, the survey highlight the relevance of environmental risk to service delivery, as shown by Sijm-Eeken [32]: some hospitals are beginning to consider how environmental degradation and climate risks inversely affect service delivery. In this sense, the greater attention on land use and building design—compared to infrastructure protection and essential clinical care—signals an emerging shift in perspective, from a singular focus on clinical process safety to broader consideration of its environmental impacts.

### Strengths and limitations

This study has several limitations. First, the small sample size and the organizational homogeneity of participating institutions—restricted to JCI-accredited hospitals—limit the generalizability of the findings to the broader and more heterogeneous Italian healthcare system. Second, not all contacted institutions responded to the questionnaire, introducing the potential for non-response bias. Third, the reliance on self-assessment, inherently associated with the utilisation of a questionnaire, mat have resulted in reporting bias the potential for non-response bias.

Despite these limitations, the study provides valuable insights into the current state of sustainability practices within a subset of high-performing hospitals, highlighting key gaps and priorities for improvement in governance, integration, and operationalization.

### Interpretation within the context of the wider literature

Although interest in sustainability in healthcare is growing, international literature still lacks detailed studies on how healthcare organizations operationalize environmental goals. The present findings echo those of Carnero and Langstaff regarding the impact of infrastructure and resource consumption, especially energy, as primary foci of sustainability interventions [33, 34].

Moreover, while existing literature emphasizes the importance of engaging all hospital personnel, including technical and maintenance staff, in sustainability initiatives, the present study reveals that leadership in sustainability efforts tends to be limited to senior management. This has clear practical implications: without distributed leadership and frontline ownership, sustainability is unlikely to influence daily clinical and operational decisions, where many high-impact behaviors occur (e.g. procurement practices, use of consumables, waste segregation). This finding directly supports the recommendation to broaden engagement beyond senior leadership and formally integrate clinical, technical, and maintenance staff into sustainability governance structures.

### Implications for policy, practice and research

The implications of our findings are threefold. First, the observed heterogeneity in governance structures and committee composition, likely reinforced by the absence of a national regulatory framework, supports the need for clearer national and regional guidance and alignment with accreditation standards. Second, the concentration of sustainability leadership within senior management, coupled with limited mechanisms for staff participation, indicates that hospitals should prioritize broader workforce engagement and distributed responsibility. Third, gaps in systematic monitoring and the limited uptake of long-term technological investments highlight the need for standardized indicators and financing strategies that support high-impact decarbonization interventions.

These findings underscore the pressing need for enhanced governance mechanisms, systematic monitoring, and increased workforce involvement to foster sustainability in Italian hospitals. It is submitted that national and regional health authorities, in collaboration with accreditation bodies such as JCI, could play a key role in setting clear sustainability goals, providing technical support and promoting best practices. Moreover, the integration of environmental sustainability into the day-to-day activities of hospitals can be facilitated by the leveraging of existing JCI structures, such as quality improvement teams.

The healthcare sector is responsible for a significant proportion of greenhouse gas emissions and environmental degradation. Consequently, there is a need to accelerate the adoption of comprehensive sustainability practices in hospitals. This is not only an issue of institutional responsibility, but also of public health.

More broadly, the findings call for a rethinking of academic research on health systems. Traditionally polarized between legal-structural analyses and clinical outcomes research, the field must evolve toward a more integrated perspective—one that embraces health, environmental, legal, and managerial dimensions. This paradigm shift aligns with an expanded vision of the One Health approach and could foster new interdisciplinary research fields and policy dialogues.

## Conclusions

The concept of environmental sustainability is progressively assuming a pivotal role within global health systems. This is primarily due to the substantial environmental impact that these systems are capable of exerting, in addition to the significant repercussions that this impact can have on human health. However, it is evident that the Italian healthcare system does not currently incorporate an assessment of environmental impact within the accreditation processes of its facilities. This aspect is instead evaluated exclusively by international accreditation organisations such as the JCI.

This study constitutes the inaugural endeavour to analyse the Italian context, with a particular focus on the implementation of assessment and intervention measures within Italian JCI-accredited structures. This is a fundamental component, particularly in the context of integrating environmental issues into healthcare assessment processes, and at the same time represents a first step towards spreading this mindset, free from specific national and international accreditation and assessment logic in the healthcare context.

Despite the heterogeneity observed in the study with regard to the implementation of practices and the level of attention accorded to these issues, the practices introduced thus far encompass elements that are transferable and adaptable across diverse professional settings, contingent upon the characteristics of the healthcare facility in question and the prevailing socio-economic and environmental context.

## Data Availability

The data underlying this article will be shared on reasonable request to the corresponding author.
